# Interaction of PCE and Chemically Modified Starch Admixtures with Metakaolin-Based Geopolymers—The Role of Activator Type and Concentration

**DOI:** 10.3390/ma18174154

**Published:** 2025-09-04

**Authors:** Stephan Partschefeld, Jasmine Aschoff, Andrea Osburg

**Affiliations:** Finger Institute of Building Materials Science, Bauhaus-Universität Weimar, 99423 Weimar, Germany; jasmine.aschoff@uni-weimar.de (J.A.); andrea.osburg@uni-weimar.de (A.O.)

**Keywords:** metakaolin, geopolymers, polycarboxylate ether, anionic and cationic starch admixtures, solubility, adsorption, alkali hydroxide solutions

## Abstract

Water-reducing admixtures are of enormous importance to adjust the workability of alkali-activated materials. Especially in geopolymers activated by highly concentrated alkaline solutions, the polycarboxylate ether (PCE) superplasticizers are less effective than in conventional cementitious systems. The aim of this study was to clarify the reasons for the lower dispersing performance of PCE and the synthesis of alternative dispersing agents based on the biopolymer starch to improve the workability of highly alkaline geopolymers. Furthermore, the focus of investigations was on the role of activator type and concentration as key parameters for geopolymer reaction and interaction of water-reducing agents. Therefore, in this study the conformation of three different types of PCE (MPEG: methacrylate ester, IPEG: isoprenol ether, and HPEG: methallyl ether) and synthesized starch admixtures in sodium and potassium hydroxide solutions (1 mol/L up to 8 mol/L) were studied. Furthermore, the dispersing performance, adsorption behavior, and influence on reaction kinetics in metakaolin-based geopolymer pastes were investigated in dependence on activator type and concentration. While the PCE superplasticizers show coiling and formation of insoluble aggregates at activator concentrations of 3 mol/L and 4 mol/L, the synthesized starch admixtures show no significant change in conformation. The cationic starch admixtures showed a higher dispersing performance in geopolymer pastes at all activator concentrations and types. The obtained adsorption isotherms depend strongly on the activator type and the charge density of the starch admixtures. The reaction kinetics of geopolymer pastes were not significantly influenced using the synthesized starch admixtures. Especially the cationic starch admixtures allow the reduction of liquid/solid ratios, which leads to higher flexural and compressive strengths.

## 1. Introduction

In recent years, the construction and building industry has been undergoing changes due to new technologies and ecological aspects. Because of the high emissions during cement manufacturing, alternative binder systems are coming more and more into focus [1]. One group of these alternative binders is alkali-activated binders. In addition to reduced emissions, they also have some improved properties compared to Ordinary Portland Cement (OPC) [2].

Geopolymers, a subgroup of alkali-activated materials, are a relatively novel material binder group that is eco-friendly and causes low-carbon emissions compared to OPC. They have a characteristic amorphous, inorganic, three-dimensional polymer structure [3]. Classically, geopolymers consist of an alumino-silicate material that is activated by an alkaline activator like sodium or potassium hydroxide solutions or alkali silicate solutions. There is also a synthesis route with acid activators, but it is less common [4]. As alumino-silicate raw material, different industrial residues or natural products can be used, for example, slag, fly ash, or calcined clays [5,6,7,8,9,10,11].

**Figure 1:**
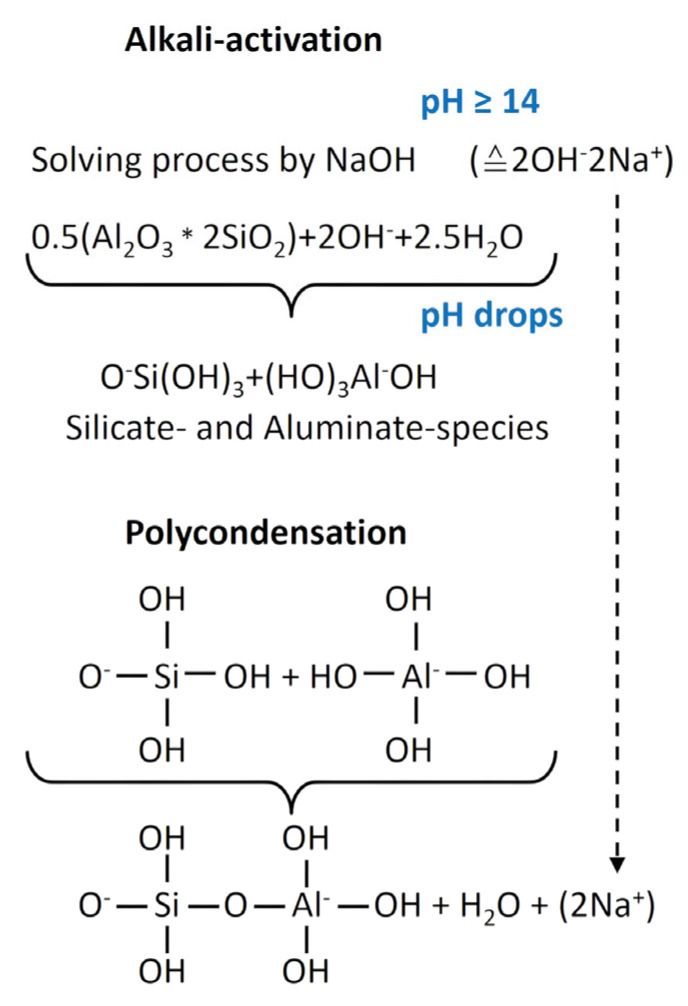
Schematic illustration of geopolymer formation by use of metakaolin and sodium hydroxide [4,16,17].

The global availability of clays makes them an excellent alumino-silicate raw material source for geopolymer manufacturing [10]. The most famous calcined clay is metakaolin. It is the anhydrous form of kaolinite (1:1 clay mineral), obtained by calcination at 500–900 °C [5,7,8,9]. There are several studies on geopolymers, where metakaolin was used by alkali activation [5,6,7,8,9,10]. Figure 1 displays the general reactions when adding an alkaline activator solution to metakaolin. The first step of geopolymer formation is a dissolution process, where the initial high pH value decreases due to the release of silicate and aluminate species. These species combine by a polycondensation reaction to form oligomers, which are the starting point to build up the geopolymer network due to inter-particle bonding, and the solidification starts [4,5,6,8].

The resulting geopolymer is distinguished by properties like great mechanical strength and fire and chemical resistance. But all properties depend on the types and amounts of raw materials and activators and other additives used, the water content, and the curing conditions [5,8,9,10]. Many geopolymer compositions suffer from insufficient workability. By the use of admixtures, the processing and other properties can be improved. Famous admixtures to improve workability are superplasticizers. These materials are water reduction admixtures that can improve the workability of the geopolymer pastes and mortars [11]. Currently, polycarboxylate ethers (PCE) as high-performance superplasticizers are state of the art. An overview of the different chemical structures of these superplasticizers is given by Plank et al. [12]. In general, PCEs are comb polymers, which consist of a negatively charged main chain of carboxylic acids and side chains of polyethylene glycol. The working mechanism of PCE is based on steric and electrostatic inhibition due to anionic groups, which can be adsorbed on positively charged mineral surfaces. The side chains protrude in the pore solution where the space requirement is limited and steric hindrance occurs [12,13]. For clay minerals, PCE interacts with surface adsorption and chemical intercalation. Intercalations mean that the side chains of the PCE form hydrogen bonds with the surfaces of the intermediate layers of the alumino-silicates. The PCE are enclosed in the intermediate layers, which reduces their performance. This phenomenon occurs mainly in uncalcined montmorillonite clays and does not occur in calcined clays [13,14].

For Portland cement systems with 30 wt.-% fly ash as a cement substitute activated by NaOH, studies on the effectiveness of PCE (MPEG type) were conducted by Marchon et al. [15]. The authors report a significant loss in flowability when using PCE in alkaline-activated cements. As a reason for this, the researchers mentioned changes in adsorption degree when using NaOH instead of water. There is a competition between the PCE and the OH^−^ in an alkaline milieu. PCE side chains did not seem to be hydrolyzed or have any other changes in PCE molecules.

For cement systems blended with calcined clays, the water demand increases compared to OPC because of the high surface of the clays. Therefore, effective water-reducing admixtures are necessary. Moghul et al. [18] used an LC^3^ cement system (limestone calcined clay cement) and an MPEG PCE as a flow agent, aiming to learn about the impact of calcined clay on the PCE. Their study reports that the PCE was not able to manage slump loss of the cementitious system. The reason for this behavior was assigned to the initial strong adsorption of the PCE on particle surfaces, so over time there is not enough reserve to adsorb when new surfaces are created. The issue is that adding more PCE was not possible because it will lead to segregation or bleeding. Another study dealing with LC^3^ cement and using PCE (HPEG and MPEG) from Sposito et al. [19] showed that HPEG PCE is slightly more effective compared to MPEG PCE because of its smaller side chains and higher anionic charge density. Furthermore, Schmid et al. [20] investigated the performance of three different PCE types (MPEG, HPEG, and IPEG) in cement systems composed of OPC and up to 50 wt.-% calcined clay (illite-smectite-rich, low kaolinite). The PCE demand increases with increasing calcined clay content due to the higher surface areas. For PCE performance, with longer side chains and higher anionic charge, the effectiveness rises. HPEG superplasticizers (high anionic charge density, long side chains) and MPEG PCE (shorter side chains) were found to be most effective in such binder systems. One main challenge when using PCE in alkali-activated binders is the ionic strength of the activator solution. Chen et al. [21] found out that with increasing ionic strength, the steric inhibition becomes inhibited due to competitive adsorption with silicate anions and strong coiling of the PCE molecules. If the ionic strength becomes too high, the PCE molecules flocculate and become insoluble. For this reason, it is important to research alternative plasticizers that are not affected that much by high alkali solutions. Similar observations were made by Tutal et al. [22]. The researchers report that ester bonding of PCE superplasticizers did not work in very basic media. Other studies confirm the observation that in high alkaline environments, ester-based superplasticizers like PCE tend to degrade and lose their effects, while superplasticizers based on ethers retain their molecular structure and remain effective [14,21,23,24,25,26]. When activating Portland cement blended with slag by alkaline sodium hydroxide or sodium silicate solution, Palacios et al. [27] also found that the chemical structure of PCE superplasticizers is modified by high alkaline milieu, leading to a modified performance compared to their effect in OPC. Nevertheless, there is no unanimous consensus on the ineffectiveness of PCE in alkali-activated materials. Due to the fact that many researchers work with different minerals (fly ash, ground granulated furnace slag, calcined clays, and other alumino-silicate sources), no clear picture of superplasticizer interaction in alkali-activated materials occurs.

For metakaolin-based binders activated by sodium silicate solution, Favier et al. [28] report only minor colloidal interaction of the suspended metakaolin particles. Therefore, the geopolymer system can be seen as non-colloidal. Hence, no deflocculating agents will work well in metakaolin-based geopolymers. Due to fewer particle interactions in pore solutions, polymer admixtures, like plasticizers, will just adsorb in a small amount on particle surfaces, and consequently polymers remain in the solution without adsorbing. These will lead to an increase in viscosity, which is not desirable. The viscosity of these geopolymers with low calcium content only depends on the viscosity of the alkaline activator. For this reason, the viscosity of the geopolymer pastes can decrease by a factor of five when changing the activator from sodium silicate to potassium silicate solution [3,28].

An alternative to commercial, petrochemical PCE superplasticizers are plasticizers based on renewable raw materials. Adequate sources for such admixtures are polysaccharides like cellulose and starch. There are several publications on the use of starch-based superplasticizers in alkaline environments. Tutal et al. [22] modified cassava starch by implementing sulfonic acid groups and reported a plasticizing effect in alkali-activated geopolymers. Vieira et al. [29] used sulfated maize starch-based admixtures for Portland cement systems, reaching a comparable dispersing performance to PCE. In addition, Crépy et al. [30] synthesized different admixtures from modified starch for use in Portland cement systems, resulting in promising results for sulfonated starches. The researchers also figured out that with a higher degree of substitution, meaning with more side chains, the effectiveness of the liquefying effect of the starches increased due to the higher repulsion between the admixture-coated particles.

The aim of this study is to gain new insights into the interaction of plasticizers based on starch in high-alkali environments compared to different PCE types. Therefore, anionic and cationic starch-based admixtures with two different charge densities, respectively, were synthesized. At first the conformation of PCE and synthesized starch admixtures in different activator solutions (1 mol/L up to 8 mol/L) was investigated. In addition, the solubility of metakaolin in the different types of activators was measured, and the dispersing performance of the starch admixtures was obtained by mini slump and rheological measurements. Adsorption experiments were performed to gain insights on particle–polymer interaction depending on the type and concentration of activator. Finally, the influence of starch admixtures on reaction kinetics and mechanical properties of geopolymer pastes and fine mortars was determined.

## 2. Materials and Methods

### 2.1. Materials

#### 2.1.1. Metakaolin

The metakaolin (MK) used in this study was provided by the NEWCHEM GmbH (Traiskirchen, Austria) and is called Metaver^®^ O. This metakaolin is approved for use in concrete in accordance with standard NF 18-513 [31]. The chemical composition was measured by ICP-OES (inductively coupled plasma optical emission spectroscope, Aktiva M, Horiba, Kyoto, Japan) after chemical digestion and is shown in Table 1. The mineral composition of the material was determined by quantitative X-ray diffraction (XRD) analysis using a Seifert XRD 3003 TT diffractometer (GE Inspection Technology, Hamburg, Germany). The quantitative phase composition was calculated by Rietveld refinement (AutoQuan 2.0, XRD Eigenmann GmbH, Karlsruhe, Germany) using 10 wt.-% zinc oxides as an internal standard. As shown in Table 1, the main phases are kaolinite (approx. 24 wt.-%) and an amorphous phase (approx. 72 wt.-%). Its particle size was obtained by laser granulometry (Laser particle analyzer, LS 230, Coulter, Indianapolis, IN, USA), and the d_10_, d_50_, and d_90_ values are given in Table 1, as well as the specific surface by BET measurement (BET Analyzer, Coulter SA 3100, Indianapolis, IN, USA). The pozzolanic reactivity of the metakaolin was determined by calorimetric investigations at 40 °C for 7 days (R^3^-Test) [32] and the modified Chapelle test according to French standard NF P18-513 [31], and the values are also given in Table 1.

#### 2.1.2. Alkaline Activators

For alkali activation of the metakaolin, two types of activators based on sodium and potassium hydroxide solutions were prepared. For each type of activator, three different concentrations (1 mol/L, 4 mol/L, and 8 mol/L) were generated by adding NaOH pellets (≥98%, Carl Roth GmbH + Co. KG, Karlsruhe, Germany) or KOH pellets (≥85%, Carl Roth GmbH + Co. KG, Karlsruhe, Germany) to deionized water. The alkaline solutions were stored in a vertical shaker until complete dissolution. Some chemical parameters of the used activators in this study are given in Table 2.

The activity coefficient, activity, and pH value of the prepared 1 mol/L, 4 mol/L, and 8 mol/L NaOH and KOH solutions could not be determined due to their high alkalinity. Therefore, thermodynamic data from [33,34] were used, and the preparation was carried out with ultrapure water to avoid contamination by other ions.

#### 2.1.3. PCE Superplasticizers

**Figure 2:**
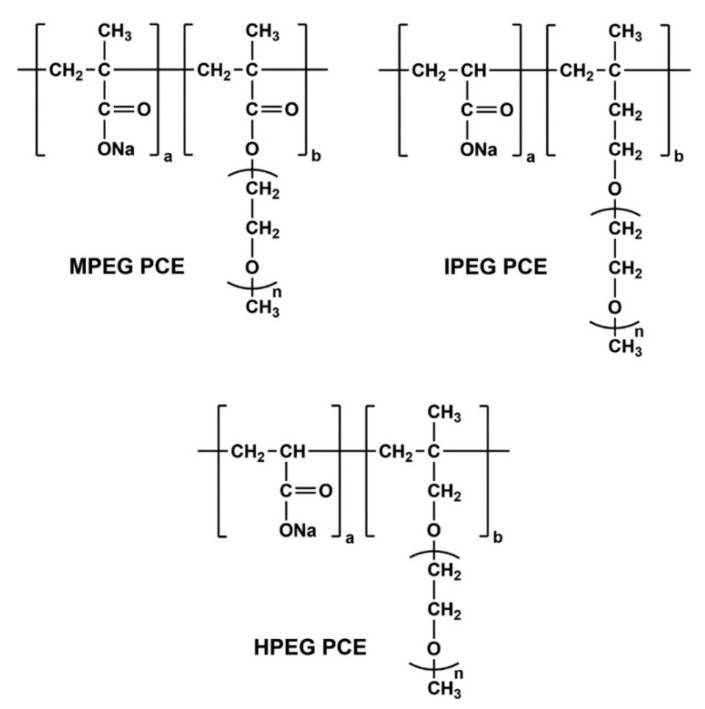
General chemical structures of the PCE superplasticizers used in this study [12].

Three different types of PCE, which were provided by MBCC Group (Master Builders Solutions Deutschland GmbH, Mannheim, Germany), were used to investigate the interaction with different alkaline activators and metakaolin. The PCE varied by their chemical architecture in the form of two ether types, HPEG PCE (methallyl ether) and IPEG PCE (isoprenol ether), and one ester type, MPEG PCE (metacrylate ester), which are shown in Figure 2.

**Figure 3:**
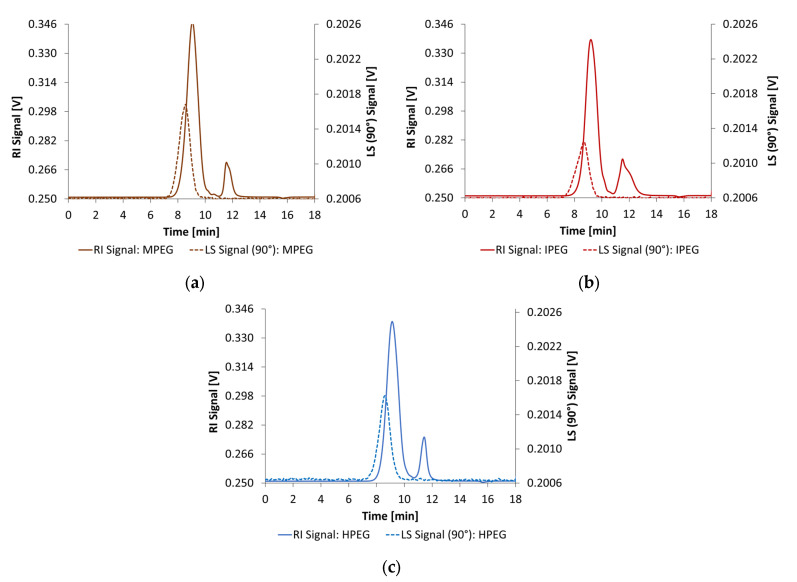
General chemical structures of the PCE superplasticizers (**a**) methacrylate ester, (**b**) isoprenol ether, and (**c**) methallyl ether used in this study [12].

Size exclusion chromatography (SEC) was utilized to determine the number-average molecular mass (Mn) and weight-average molecular mass (Mw) and the polydispersity index (PDI) of PCE superplasticizers. An AF2000 MultiFlow FFF System (Postnova Analytics GmbH, Landsberg am Lech, Germany) was equipped with a Suprema Max linear XL column (PSS Polymer Standards Service GmbH, Mainz, Germany) for anionic polymers. The particle size of the column materials was 10 µm. A multi-angle light scattering detector (MALS) and a refractive index detector (RI) were used to determine the sample fractions. While the MALS was used to obtain the absolute molecular weight, the RI was calibrated using PMA (polymethylacrylate) calibration standards (ReadyCal-Kit PMA, 1200 Da to 1000 kDa, PSS Polymer Standards Service GmbH, Mainz, Germany) for the PCE superplasticizers. A 0.05 wt.-% NaN_3_ solution was used as eluent. For the sample preparation, 5 mg of each PCE was dissolved in 10 mL of the eluent. The flow rate was set to 1.0 mL/min. To calculate the molecular weight of the PCE superplasticizers, the dn/dc value (refractive index increment) was measured by direct injection without separation by column and set to 0.170 mL/g. The amounts of anionic charges were determined using a PCD 04 particle charge detector (Mütek Analytic, Herrsching, Germany). The polymer solutions were prepared in deionized water with a concentration of 0.01 wt.-%. For polyelectrolyte titration, a 0.001 N polydiallydimethylammoniumchloride (Poly-DADMAC, Mütek Analytic, Herrsching, Germany) solution was used as a titrant. The molecular parameters of the PCE are illustrated in Table 3. The SEC chromatograms of all PCE samples are shown in Figure 3. The first peak correlates to the polymer fraction, while the second peak is assigned to macromonomer residuals.

#### 2.1.4. Preparation of Anionic and Cationic Starch Admixtures

Modified starch admixtures are used in this study to investigate their liquefying effect on metakaolin-based geopolymers. A short-chained cassava starch (Ingredion GmbH, Hamburg, Germany) was used as basic starch for synthesis of anionic starch admixtures (SES) and cationic starch admixtures (KS).

For synthesis of anionic starches, 30 g of the basic starch was suspended in 270 mL of IPA (isopropyl alcohol, ≥99.9%, Carl Roth GmbH + Co. KG, Karlsruhe, Germany) using a 500 mL round-bottom flask. A magnetic stirrer with a heat function (IKA RCT digital, IKA-Werke GmbH + Co. KG, Staufen, Germany) was used to heat the suspension from RT (room temperature) to 30 °C under continuous stirring. Then, 15 g of sodium hydroxide pellets (≥98%, Carl Roth GmbH & Co. KG, Karlsruhe, Germany) were added to the suspension. After dissolution, a 25 wt.-% sodium vinyl sulfonate solution (SVS, Sigma-Aldrich, Inc., Darmstadt, Germany) was added dropwise for 10 min. To implement two different amounts of anionic charges, the amount of SVS was doubled from 20 g to 40 g. Subsequently, the suspension was heated to 60 °C and stirred for 4 h.

**Figure 4:**
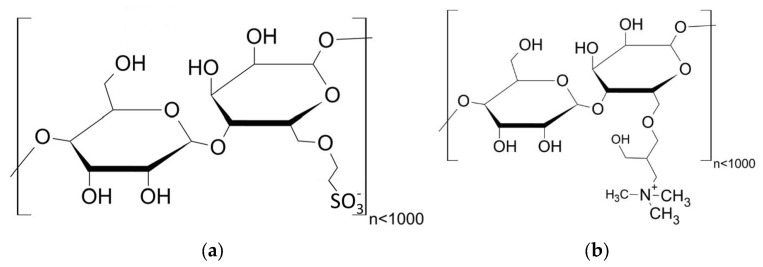
General structure of the synthesized anionic (**a**) and cationic (**b**) starch-based admixtures.

For synthesis of cationic starches, 30 g of the basic starch was suspended in 270 mL of IPA (≥99.9%, Carl Roth GmbH + Co. KG, Karlsruhe, Germany) using a 500 mL round-bottom flask. Then, 15 g of NaOH pellets (≥98%, Carl Roth GmbH + Co. KG, Karlsruhe, Germany) were added, and the suspension was cooled down to 10 °C by transferring the round-bottom flask to a crystallization dish, which was positioned on a magnetic stirrer and filled with a mixture of water and ice. Subsequently, 50 wt.-% (3-chloro-2-hydroxypropyl) trimethylammonium chloride (CHPTAC) was added dropwise, and the suspension was stirred for 4 h at 10 °C. To adjust two different amounts of cationic charges, the amount of CHPTAC was doubled from 7.5 g to 15 g. After 4 h of stirring, the IPA of the suspensions was removed using a rotary evaporator. The solid starch admixtures were diluted in 200 mL of deionized water and neutralized with 37 wt.-% hydrochloric acid. Each starch admixture was dialyzed for 8 h using dialysis tubes with a cutoff of 1000 Da to remove unreacted chemicals from the starch admixtures. The general molecular structures of the two anionic (SES-1 and SES-2) and two cationic starch admixtures (KS-1 and KS-2) are shown in Figure 4.

To determine the molecular weight of the anionic starch admixtures, the same procedure as for the PCE was used. For the cationic starch admixtures, a Novema Max column (PSS Polymer Standards Service GmbH, Mainz, Germany) was used. A 0.5 wt.-% formic acid with 0.05 wt.-% of NaN_3_ was used as eluent, and pullulan calibration standards (ReadyCal-Kit Pullulan, 180 Da to 805 kDa, PSS Polymer Standards Service GmbH, Mainz, Germany) were used to calibrate the RI. For all starch admixtures, the dn/dc value was set to 0.140 mL/g.

**Figure 5:**
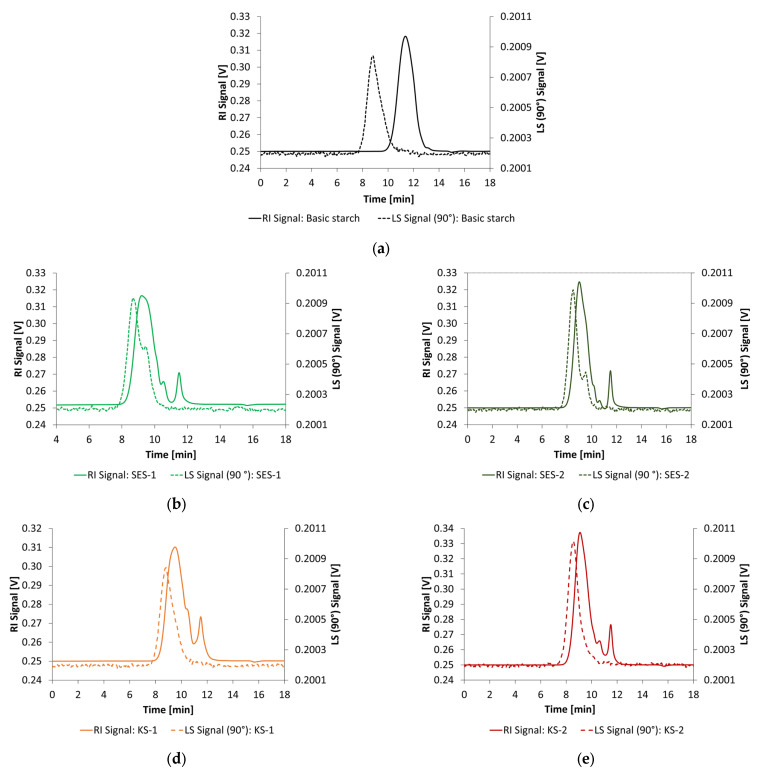
SEC chromatograms of the basic starch (**a**) and the synthesized anionic (**b**) SES-1, (**c**) SES-2 and cationic ((**d**) KS-1, (**e**) KS-2) starch admixtures.

Chemical modification results in an increase in molecular weight by increasing the number of charges. This effect cannot be explained solely by the substitution of hydrogen atoms on the hydroxide groups. Rather, it can be assumed that the molecular structure of the starches is expanded by the implemented charges. This determines an apparently larger molecular weight because the starch molecules no longer fit into the small pores of the separation column. As shown in Table 4, by doubling the concentration of modifying reagents, the implemented number of charges was increased by the factor of 2.5 for SES and 2.1 for the KS starch admixtures. To determine the number of anionic charges, the same procedure as for the PCE was used. For the cationic starch admixtures, the titrant was changed to 0.001 N sodium polyethylene sulfonic acid (PES-Na, Mütek Analytic, Herrsching, Germany) solution. Figure 5 shows the SEC chromatograms of the basic starch and the synthesized starch admixtures.

#### 2.1.5. Fine Aggregates

A granite crushed sand (Granitwerk Fischer GmbH & Co. KG, Wurzbach, Germany) with a grain size of 0–0.5 mm was used to prepare geopolymer fine mortars to investigate the influence of admixtures on their mechanical properties. This sand was selected to compensate for shrinkage during the geopolymer reaction and dehydration, which caused cracks [35]. The chemical and mineralogical composition is given in Table 5 as well as the physical parameters.

### 2.2. Methods

#### 2.2.1. Dynamic Light Scattering

The dynamic light scattering (DLS) method was used to investigate the interaction of the PCE superplasticizers and starch admixtures with the alkaline activators. Therefore, a ZetaSizer Nano S (Malvern Panalytical GmbH, Kassel, Germany) was used. The focus of the investigations was to discover the activator concentration at which coiling and ultimately insoluble agglomerates are formed, which was reported for PCE by Chen and Plank [21]. Therefore, 1 g/L of each PCE was diluted in deionized water and different concentrations of alkaline activators (NaOH and KOH: 1 mol/L, 2 mol/L, 3 mol/L, 4 mol/L, and 8 mol/L). The synthesized starch admixtures were treated with different activators in the same way. All DLS measurements were performed 3 times, and an average Rh value was calculated.

#### 2.2.2. Solubility of Metakaolin in Activator Solutions

The first step of the geopolymer reaction is the dissolution of aluminate and silicate species on the surface of the metakaolin particles caused by an alkaline activator. The amount and kinetics of the dissolution process depend on the type and concentration of the alkaline activator. Therefore, solubility investigations were performed using two types of activators, sodium and potassium hydroxide solutions in concentrations of 1 mol/L, 4 mol/L, and 8 mol/L. To minimize the precipitation of reaction products, a high liquid-to-solid ratio (l/s) of 20 was used. The preparation of the samples followed the same manner for each type of activator and concentration. First, polypropylene centrifuge tubes were filled with 5 g of metakaolin and mixed with 20 mL of the respective alkaline activator. The filled centrifuge tubes were stored in a vertical shaker and removed after 15, 30, 60, 120, 240, 480, and 1440 min, centrifuged, and the supernatant collected with a syringe and filtered with a 0.45 µm syringe filter. Subsequently, 1 mL of the filtered supernatant was transferred to 24 mL of 1.0 wt.-% HNO_3_ to avoid precipitation and carbonation. The time-dependent amount of dissolved silicon and aluminum was determined using ICP-OES (inductively coupled plasma optical emission spectroscope, Aktiva M, Horiba, Kyoto, Japan).

#### 2.2.3. Mini Slump and Flow Table Test

To identify the influence of the synthesized starch admixtures on the workability of geopolymer pastes, the mini slump test was used. The procedure is described by Tan et al. [36]. Therefore, the pastes were mixed from metakaolin, alkaline solution, and admixture. In addition, a reference for each alkaline solution was mixed without admixture. The paste was filled in the cone, which was placed on a flow table. Then, the cone was removed, and the spread flow of the paste was measured two times in different directions by an electronic caliper, and a mean value was calculated. Afterwards, the paste was shocked 15 times, and the spread was measured again in two different directions, and another mean value was calculated. The two concentrations, 0.7% and 1.5% bwob (by weight of binder), of the anionic and cationic starches were tested. Each mini slump and flow table test was performed twice, and an average value was calculated.

#### 2.2.4. Rheological Tests by Rotational Viscosimeter

Changes in the rheological behavior of pastes by the use of the modified starch admixtures were detected by measuring the dynamic viscosity and shear stress by a rotation viscosimeter (Rheotec^®^ Brookefield DV III-ultra, Middleboro, MA, USA) with an SC-4 29 spindle. The measurement followed a defined shear profile: At first the pastes were pre-shared for 3 min with a shear rate of 120 rpm; after that the shear rate was reduced every 30 s to 100, 80, 60, 40, 20, 10, 5, 2, and 1 rpm. During the investigations, a measuring value is recorded every 5 s. The measured values recorded at each shear rate (5 individual measurements) were calculated to an average value, and the flow curves were obtained. For comparison of the rheological measurements with the mini slump, the same paste compositions were chosen.

#### 2.2.5. Adsorption Experiments

**Figure 6:**
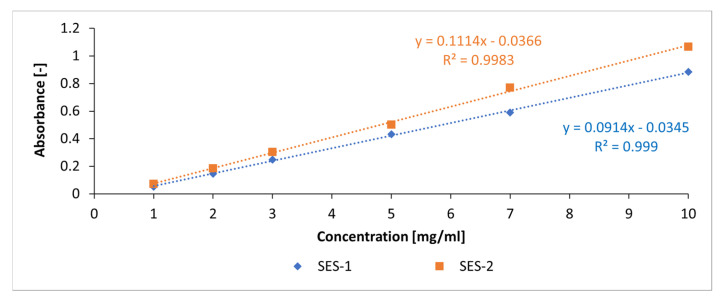
Example of calibration curves of the anionic starch samples SES-1 and SES-2 in 4 mol/L KOH solution.

In order to obtain information on whether and in what quantity the starch admixtures adsorb on the surfaces of the metakaolin particles, adsorption experiments were performed using a UV–vis spectrometer (SPECORD^®^, Analytik Jena, Jena, Germany) based on the method of Perche [37]. Due to the presence of UV-active molecular groups (sulfonic acid groups and ammonium groups), a wave range from 190 nm to 350 nm was selected. The reference sample for all measurements was deionized water. The first step of this method is the creation of calibration curves for each admixture dissolved in the alkaline activators (NaOH and KOH: 4 mol/L, 8 mol/L). Therefore, each sample of admixture was diluted in a concentration range with the steps 0 mg/mL, 1 mg/mL, 2 mg/mL, 3 mg/mL, 5 mg/mL, 7 mg/mL, and 10 mg/mL in each activator. The absorbance at each concentration was measured at a wavelength, which provides a linear relationship to the selected concentration. An example of a calibration curve is given in Figure 6.

To determine the adsorption of the starch admixtures on metakaolin, both were mixed in a concentration range with the steps: 0.2 wt.-%, 0,5 wt.-%, 0.7 wt.-%, and 1.0 wt.-% related to the metakaolin. The liquid-to-solid ratio was adjusted to 1.15 (4 mol/L KOH), 1.35 (8 mol/L KOH), 1.35 (4 mol/L NaOH), and 1.4 (8 mol/L NaOH). Each sample was filled in tubes and shaken for 15 min (vertical shaker KS 250, IKA-Werke GmbH & Co. KG, Staufen, Germany) and then centrifuged for 5 min at 11.5 G (Centrifuge 5804 R, Eppendorf SE, Hamburg, Germany). The supernatant was taken with a 3 mL syringe and filtered through a 0.45 µm polystyrene filter. Each sample was measured at the same wavelength of the corresponding calibration curve. The admixture content in the supernatant was calculated according to Equation (1). The amount of adsorbed admixture was calculated according to Equation (2).

Equation (1): Calculation of admixture content in the supernatant.

C_i_ = (A − n)/m (1)

C_i_: concentration of admixture in supernatant [mg/mL];

A: measured absorbance [-];

n: axis segment of calibration curve;

m: slope of calibration curve [mL/mg].

Equation (2): Calculation of the amount of adsorbed admixture.

C_adsorb._ = (C_0_ − C_i_) × (l/s) (2)

C_adsorb._: concentration of adsorbed admixture [mg/g_MK_];

C_0_: initial concentration [mg/mL];

l/s: liquid to solid ratio [mL/g].

Each adsorption measurement was determined twice, and an average value was generated.

#### 2.2.6. Influence of Admixture on Geopolymer Reaction

To determine the influence on the reaction kinetics through the addition of the starch admixtures to the geopolymer pastes, calorimetric investigations were carried out with an isothermal heat flow calorimeter (mc cal^®^, C3-Prozesstechnik, Gieboldehausen, Germany) at 20 °C. The heat rate was recorded for a period of 2 days. The samples were mixed outside the calorimeter, then the sample container was quickly closed, and the measurement started. The cumulative heat was calculated by integration of the heat rate by time.

#### 2.2.7. Mechanical Properties

The influence of the synthesized starch admixtures on the properties of the hardened binders, such as bulk density, flexural strength, and compressive strength, was also analyzed. Therefore, a mortar was mixed from metakaolin, alkali hydroxide solution, admixture, and granite crushed sand. The binder-to-sand ratio was adjusted to 1.0 for all investigated compositions. The l/s value varied depending on activator concentration. Generally, the l/s ratio was reduced in comparison to the reference composition by 0.1 due to the dispersing performance of the synthesized starch admixtures. Mortars were mixed using an electric stirrer with a rotation speed of 500 rpm. For each alkaline activator (KOH and NaOH: 1 mol/L, 4 mol/L, 8 mol/L), a reference sample without the addition of admixture was prepared. Six micro prisms (10 mm × 10 mm × 60 mm) were produced for each composition and cured for 28 days at RT (room temperature). After time, the dimensions and weights of the samples were measured to calculate their bulk density. Subsequently, the flexural strengths were determined using TIRAtest 28100 (TIRA GmbH, Schalkau, Germany), and the compressive strength was obtained at the two fragments of the samples. Finally, the flexural strength was determined as the average value from three test specimens and the compressive strength as the average value from 6 fragments of them. The bulk density is also an average value of 3 specimens.

## 3. Results and Discussion

### 3.1. Size Analysis of the PCE and Synthesized Starch Admixtures in Alkaline Activators

**Figure 7:**
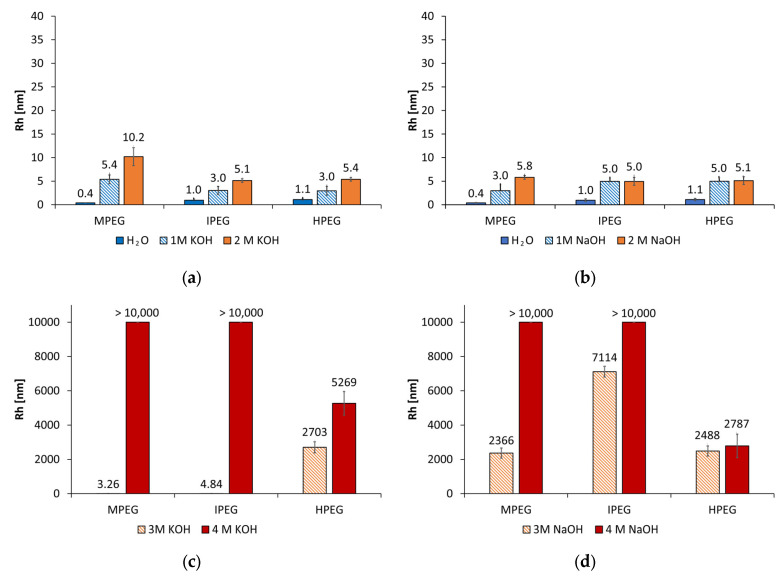
Rh values of the MPEG, IPEG, and HPEG PCE in (**a**) water, 1 mol/L KOH, and 2 mol/L KOH; (**b**) water, 1 mol/L NaOH, and 2 mol/L NaOH; (**c**) 3 mol/L KOH and 4 mol/L KOH; and (**d**) 3 mol/L NaOH and 4 mol/L NaOH.

Alkali-activated binders are characterized by a high pH value and correspondingly high ionic strength of their pore solution. This is due to the high concentration of NaOH or KOH required for activation of reactive alumosilicates [7,38]. Previous literature reported that especially PCE superplasticizers are sensitive to solubility in high alkaline environments [21,39]. For this reason, it was initially of interest to examine how the conformation of conventional PCE superplasticizers changes depending on the activator concentration. The dynamic light scattering (DLS) measurements were performed to determine the hydrodynamic radius Rh in dependence of the type of PCE as well as the concentration and type of alkaline activators. Figure 7 shows the Rh values of the MPEG, IPEG, and HPEG PCE in water and different concentrations of KOH and NaOH (1 mol/L, 2 mol/L, 3 mol/L, and 4 mol/L).

While all PCE show a small size between 0.4 nm and 1.1 nm in water, the Rh values increased slightly in the range of 3 nm to 6 nm using 1 mol/L and 2 mol/L of the alkaline activators. The differences between whether NaOH or KOH is used are negligible. At an activator concentration of 3 mol/L, the hydrodynamic radius of HPEG PCE in KOH and NaOH activators increases at approx. the same rate up to 2703 nm and 2488 nm. In comparison, the MPEG PCE and IPEG PCE show low Rh values in the range of 3 nm and 5 nm in KOH and a massive increase in NaOH activator in the range of approx. 2400 nm and 7100 nm. This indicates that the coiling effect described by Chen and Plank [21] depends on a limiting concentration and type of activator, which was identified in a range of 3 mol/L to 4 mol/L for MPEG and IPEG PCE in KOH and 3 mol/L for all investigated PCE in the NaOH activator. At a concentration of 4 mol/L, the MPEG and IPEG PCE form insoluble aggregates with Rh values larger than 10,000 nm. In contrast, the HPEG PCE showed the coiling at lower concentrations and formation of smaller insoluble aggregates of approx. 5270 nm. As a result, it can be concluded that PCE are unsuitable for use as superplasticizers in alkali-activated geopolymers due to coiling and the formation of insoluble aggregates in typical alkaline activator concentrations starting at 4 mol/L [7,38,40], whether in potassium or sodium hydroxide solution.

**Figure 8:**
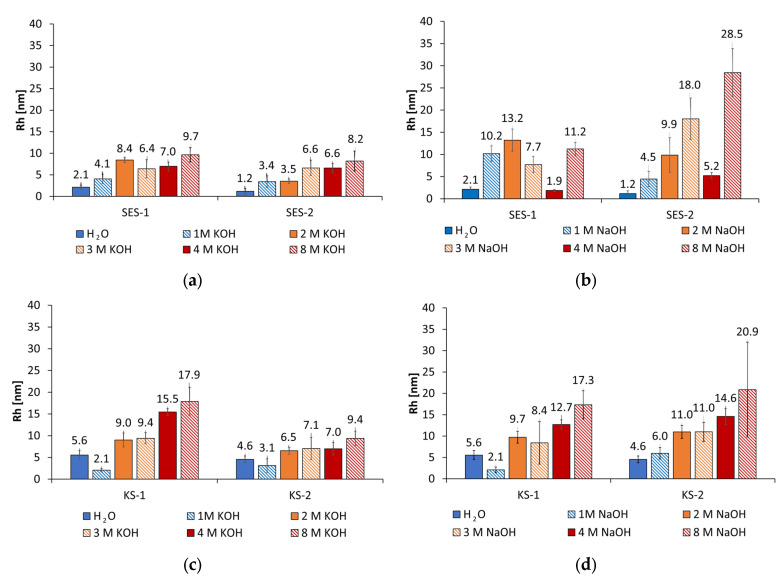
Rh values of anionic starch admixtures (**a**) in water and different concentrations of KOH and (**b**) NaOH, and cationic starch admixtures in (**c**) different concentrations of KOH and (**d**) NaOH.

The anionic and cationic starch additives show no tendency to coiling or formation of insoluble aggregates, which is presented in Figure 8. Especially in KOH activators and concentrations in the range of 1 mol/L to 8 mol/L, small Rh values are obtained in the range of 1 nm to 8 nm for SES-1 and SES-2. The Rh values of the cationic starch admixtures KS-1 and KS-2 are also small, in the range of 2 nm to 18 nm. It is obvious that the more highly charged polymers show slightly lower Rh values for both the anionic and cationic starches in KOH. In NaOH activator solutions the Rh values of the anionic starch admixtures are slightly higher than that in KOH, especially at activator concentrations of 8 mol/L. For the cationic starch admixtures, the Rh values of KS-2 are approx. twice as high in NaOH than in KOH from concentrations of 2 mol/L to 8 mol/L.

### 3.2. Solubility of Metakaolin in the Alkaline Activator Solutions

**Figure 9:**
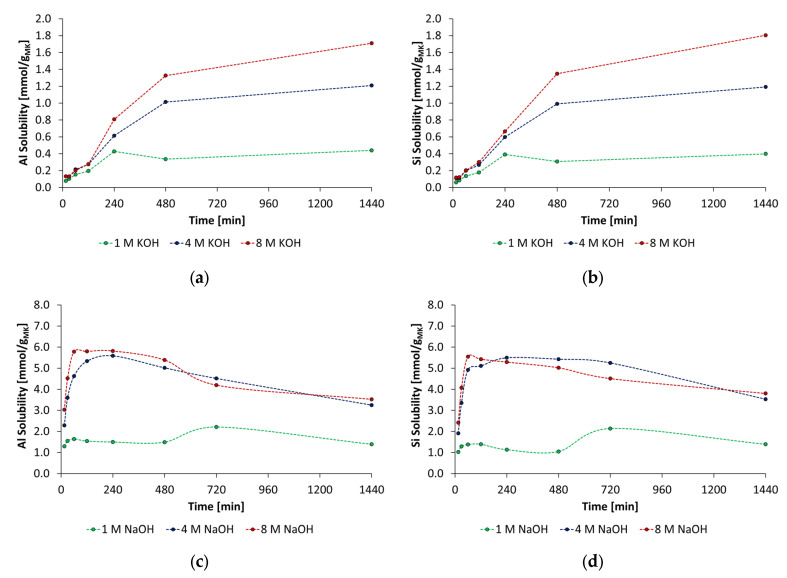
Solubility of metakaolin in KOH solutions: (**a**) aluminum solubility and (**b**) silicon solubility. Solubility of metakaolin in NaOH solutions: (**c**) aluminum and (**d**) silicon solubility (concentration: 1 mol/L, 4 mol/L, 8 mol/L, l/s = 20).

The solubility of the used metakaolin is an important parameter to select a suitable concentration of the activator. As shown in Figure 9, the solubility of metakaolin depends strongly on the type and concentration of alkaline activators. While the solubility in both 1 mol/L NaOH and 1 mol/L KOH is remarkably low, the solubility increases with the use of 4 mol/L and 8 mol/L, which was also found by Werling et al. [40,41]. The metakaolin showed congruent dissolution behavior independent of the type and concentration of the used alkaline activators. In KOH solution, maximum Al (aluminum) and Si (silicon) solubility of 1.71 mmol/g_MK_ and 1.8 mmol/g_MK_ was reached at 24 h. The highest increase in solubility was detected from 2 h to 8 h, from 0.28 mmol/g_MK_ up to 1.33 mmol/g_MK_. Subsequently, the increase in solubility is significantly lower. The solubility of metakaolin in NaOH solutions is remarkably higher in comparison to KOH solutions. The highest increase in Al and Si solubility was detected directly after contact with the NaOH activator. The maximum solubility of metakaolin of 5.6 mmol/g_MK_ (Al) and 5.5 mmol/g_MK_ (Si) was measured at 4 h for the 4 mol/L NaOH and 5.8 mmol/g_MK_ (Al) and 5.3 mmol/g_MK_ (Si) for the 8 mol/L NaOH. Then, the solubility decreased to values between 3.2 mmol/g_MK_ and 3.8 mmol/g_MK_ of Si and Al. In general, it was found that the Al and Si solubility of metakaolin in NaOH is significantly higher than in KOH, and the time-dependent progress of dissolution varies significantly. The amount of soluble Al and Si depending on activator concentration showed that concentrations of at least 4 mol/L and higher are required to dissolve sufficient amounts of metakaolin. Consequently, PCE superplasticizers in particular are unsuitable for geopolymers in combination with such activators due to the formation of insoluble aggregates, which was shown in Section 3.1. before. For this reason, only the synthesized anionic and cationic starch admixtures are considered in further investigations.

### 3.3. Dispersing Performance of the Synthesized Starch Admixtures

**Figure 10:**
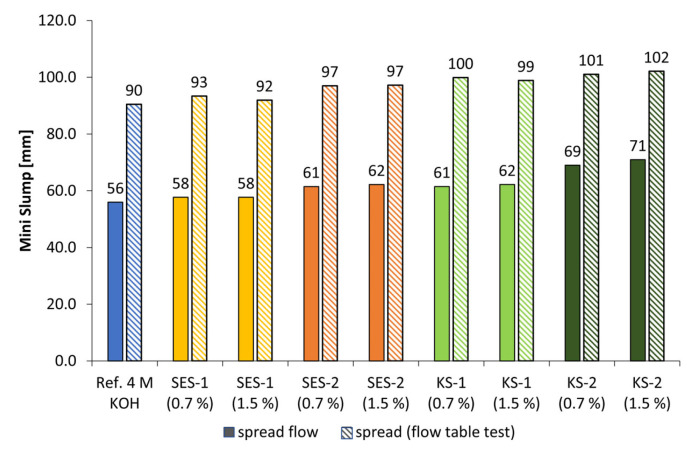
Mini slump of SES and KS admixtures (0.7% and 1.5% bwob) in geopolymer pastes activated by 4 mol/L potassium hydroxide solution (l/s = 1.0).

**Figure 11:**
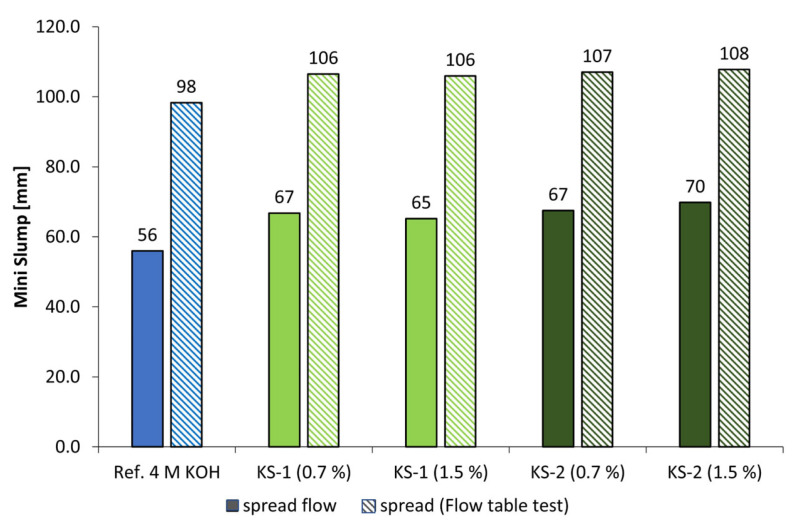
Mini slump of KS admixtures (0.7% and 1.5% bwob) in geopolymer pastes activated by 4 mol/L sodium hydroxide solution (l/s = 1.1).

Figure 10 and Figure 11 display the spread flow and spread after flow table test of alkali-activated metakaolin pastes with 4 mol/L of KOH (l/s = 1.0) as well as the influence of anionic and cationic starch admixtures in concentrations of 0.7% and 1.5% bwob. The l/s values had to be adjusted to obtain comparable reference (Ref.) spread flows. While the low-charged anionic admixture SES-1 caused a minimal increase in spread flow of approx. 3% in 4 mol/L KOH at both concentrations, SES-2 with twice the anionic charge density caused an increase in spread flow between 9% (0.7% bwob) and 10% (1.5% bwob). In addition, the anionic starch admixtures showed no influence in geopolymer pastes activated by NaOH solutions. In contrast, the cationic starch admixtures are more effective in both alkaline activators, especially the higher charged sample KS-2, with an increase in spread flow of approx. 19% (0.7% bwob) and 21% (1.5% bwob) in 4 mol/L KOH. The spread flow of the pastes activated by 4 mol/L NaOH (Figure 11) is increased in the range of approx. 14% to 20%, where KS-2 is also the most effective admixture.

**Figure 12:**
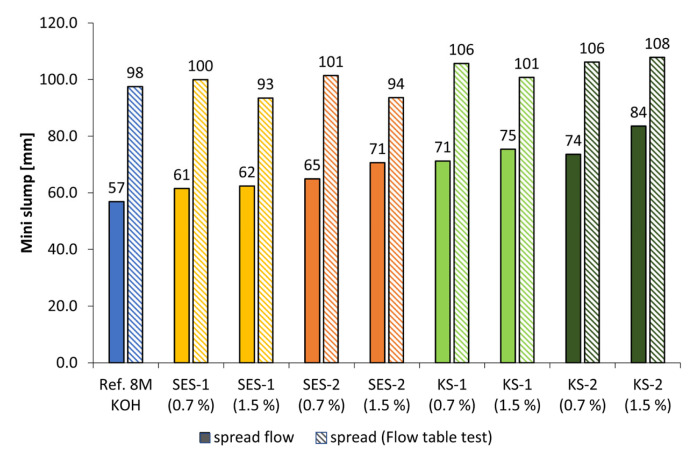
Mini slump of SES and KS admixtures (0.7 and 1.5% bwob) in geopolymer pastes activated by 8 mol/L potassium hydroxide solution (l/s = 1.1).

**Figure 13:**
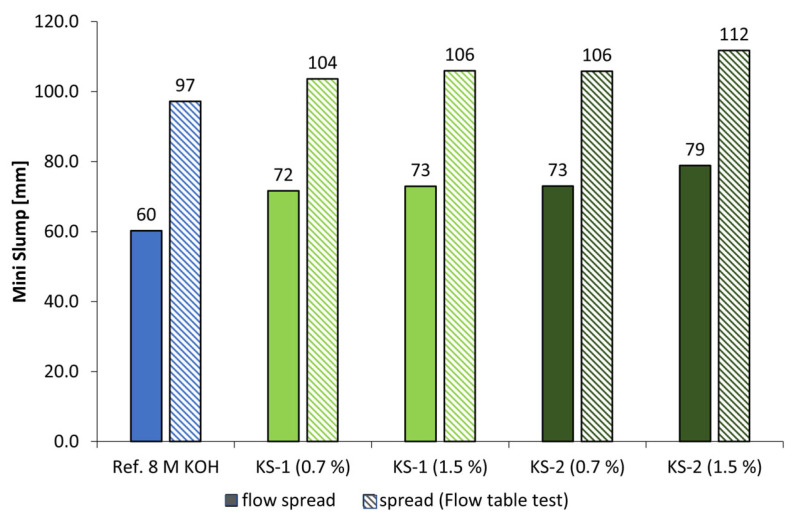
Mini slump of KS admixtures (0.7 and 1.5% bwob) in geopolymer pastes activated by 8 mol/L sodium hydroxide solutions (l/s = 1.2).

The spread flow and spread after flow table test of pastes with increased activator concentration to 8 mol/L for both activators are given in Figure 12 and Figure 13. The l/s ratio was adjusted to 1.1 for KOH and 1.2 for NaOH. The anionic starch admixture SES-1 caused an increase in spread flow of 7.5% (0.7% bwob) and approx. 9% (1.5% bwob) by use of KOH. The higher charged sample SES-2 caused a higher increase of 12.5% (0.7% bwob) and 19.5% (1.5% bwob). The same tendency was found for the cationic samples. By increasing the admixture concentration, the spread flow increased in range between 20% and 32%. For both variants of starch admixture, it was found that a higher charge density leads to a higher increase in spread flow. The highest values were reached by the cationic starch admixture KS-2. The spread flow of pastes activated by 8 mol/L of NaOH increased in the range of approx. 16% and 23.5%, depending on admixture concentration. Here, the KS-2 sample was to be the most effective admixture in the liquefaction of geopolymer paste.

**Figure 14:**
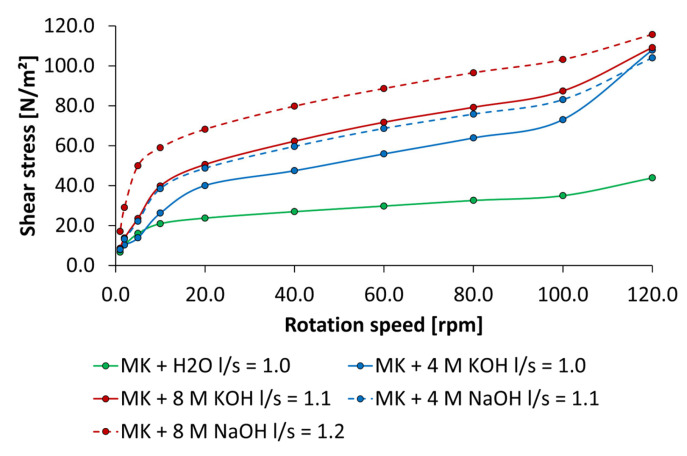
Flow curves of metakaolin pastes using pure water and 4 mol/L and 8 mol/L KOH and NaOH.

Rheological measurements of metakaolin pastes activated by the alkaline activators at concentrations of 4 mol/L and 8 mol/L and water are shown in Figure 14. Generally, all investigated pastes exhibit structurally viscous or pseudoplastic flow behavior independent of the type of activator and its concentration. The lowest shear stress was determined for the paste mixed by water at an l/s ratio of 1.0. When the alkaline activators are used, the shear stress determined at each rotation speed is noticeably higher, which corresponds to an increase in consistency. The comparison of pastes mixed with alkaline activators shows that the flow curves with KOH are lower than those with NaOH, although the l/s value for NaOH was increased by 0.1 at both concentrations. Curiously, the flow curve of metakaolin paste mixed by 4 mol/L NaOH corresponds to the flow curve mixed by 8 mol/L KOH at the same l/s ratio. At the lowest rotation speed of 1 rpm, the paste containing water showed the lowest yield stress of 6.6 N/m^2^, followed by 4 mol/L KOH with 7.8 N/m^2^ and 8 mol/L KOH with 8.5 N/m^2^. The highest value of 17 N/m^2^ was reached by 8 mol/L of NaOH at the highest l/s ratio of 1.2. Overall, metakaolin pastes activated by NaOH have higher consistencies than those activated by KOH at the same concentrations, which was also found by [3,38,42].

**Figure 15:**
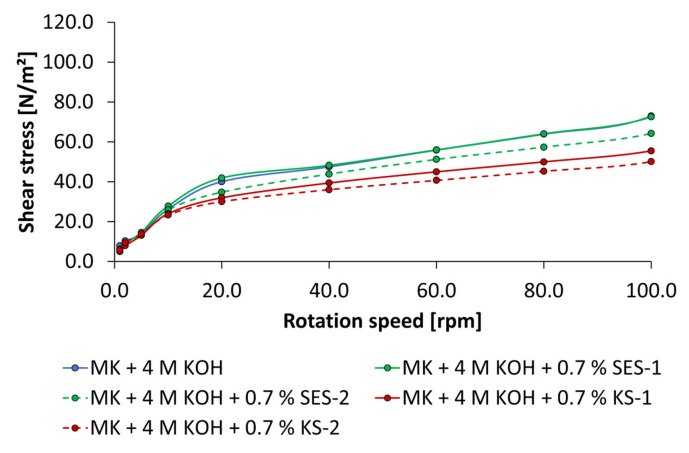
Flow curves of anionic and cationic starch admixtures (0.7% bwob) of geopolymer pastes activated by 4 mol/L KOH.

**Figure 16:**
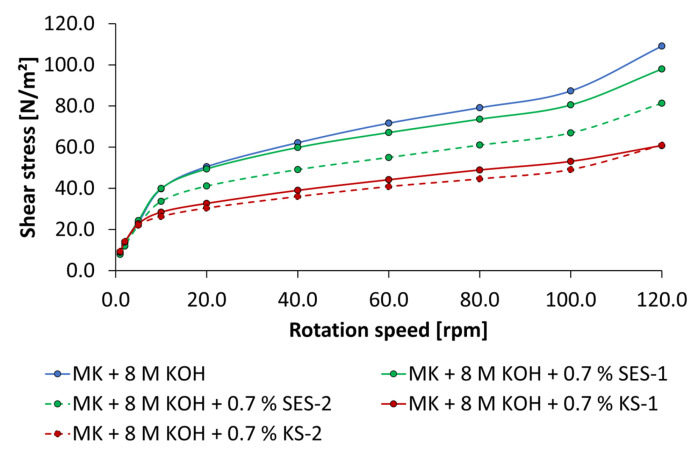
Flow curves of anionic and cationic starch admixtures (0.7% bwob) of geopolymer pastes activated by 8 mol/L KOH.

The influence of anionic and cationic starch admixtures on flow curves of geopolymer pastes activated by 4 mol/L and 8 mol/L of KOH are given in Figure 15 and Figure 16. The concentration of admixture was adjusted to 0.7% bwob, which was also used in mini slump tests. In comparison to the mini slump test, the same tendencies in liquefication of geopolymer pastes were found. The anionic sample SES-1 has a minor effect on flow curves. The higher charged sample SES-2 caused a slight reduction in shear stress at rotation speeds of 10 rpm and above. The highest liquefaction effects are determined by the cationic starch admixtures independent of KOH concentration. Especially the high-charged sample KS-2 is most effective. It should be emphasized that noticeable liquefaction effects only occur at rotation speeds of 5 rpm and above. As a result, a high yield stress must be applied to get the pastes to flow independent of the addition of admixture.

**Figure 17:**
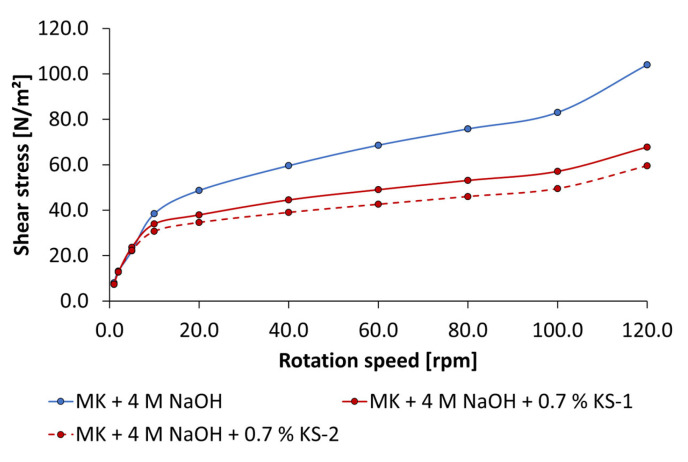
Flow curves of cationic starch admixtures in geopolymer pastes activated by 4 mol/L of NaOH (0.7% bwob).

**Figure 18:**
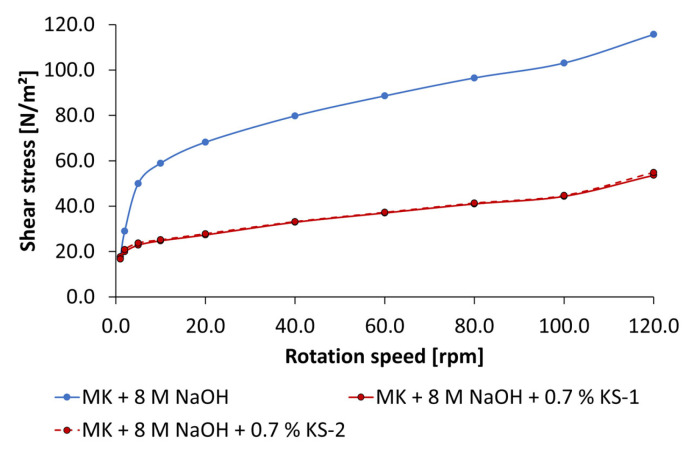
Flow curves of cationic starch admixtures in geopolymer pastes activated by 8 mol/L of NaOH (0.7% bwob).

Due to the fact that just the cationic starch admixtures show an increase of spread flow in NaOH-activated geopolymer pastes, the influence of KS-1 and KS-2 on flow behavior is presented in Figure 17 and Figure 18. The dispersing performance differs depending on activator concentration. Using 4 mol/L KOH, a reduction in shear stress occurs only at rotation speeds of 10 rpm and above. KS-2 with higher cationic charge density is more effective. In contrast, using 8 mol/L NaOH, the reduction in shear stress starts at 2 rpm and above. While the dispersing performance in pastes activated by 4 mol/L of NaOH differs between KS-1 and KS-2, in pastes activated by 8 mol/L of NaOH, both KS samples show the same dispersing performance, which is quite higher.

### 3.4. Adsorbtion Results of the Synthesized Starch Admixtures on MetakaolinParticles

**Figure 19:**
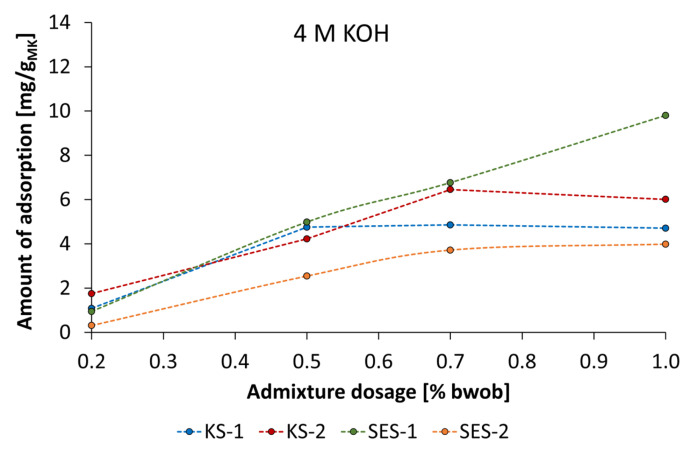
Adsorption isotherms of anionic and cationic starch admixtures of geopolymer pastes activated by 4 mol/L KOH.

Typically, consistency-influencing additives such as PCE and polycondensate-based superplasticizers (sulfonated β-naphthalene-formaldehyde polycondensates and sulfonated melamine-formaldehyde polycondensates) are surface-active substances that cause electrostatic and steric repulsion between the mineral particles by adsorption on particle surfaces [43,44,45]. To investigate the adsorption behavior of the synthesized starch admixtures, adsorption isotherms were determined depending on activator type and concentration. Figure 19 presents the adsorption isotherms of the anionic and cationic starch admixture in geopolymer pastes activated by 4 mol/L KOH at various dosages.

The anionic starch samples SES-1 and SES-2 show quite different adsorption behavior. While the amount of adsorption for SES-1 increased almost in linear progression (Freundlich-type adsorption isotherm [46]) by increasing admixture dosage, the SES-2 showed significantly lower amounts of adsorption and produced a Langmuir-type adsorption isotherm [47]. This type of adsorption was even produced by cationic starch samples, which is characterized by an increase in adsorption until its levels reach a plateau. This point typically signifies complete surface coverage and saturated adsorption. The comparison of levels of adsorption of SES-1 and SES-2 allows the conclusion that the higher charge density leads to a remarkably lower amount of adsorption. Especially at a concentration of 1% bwob, the amount of adsorption differs by a factor of 2.4. In contrast, the cationic samples KS-1 and KS-2 showed a reversed order in terms of charge density. While KS-1 reached the adsorption plateau at an admixture dosage of 0.5% bwob, the KS-2 sample reached the plateau at 0.7% bwob. The combination of dispersing performance and adsorption experiments supplies a consistent view for KS-1 and KS-2. The higher dispersing performance of KS-2 is due to a higher amount of adsorption by a factor of 1.3.

**Figure 20:**
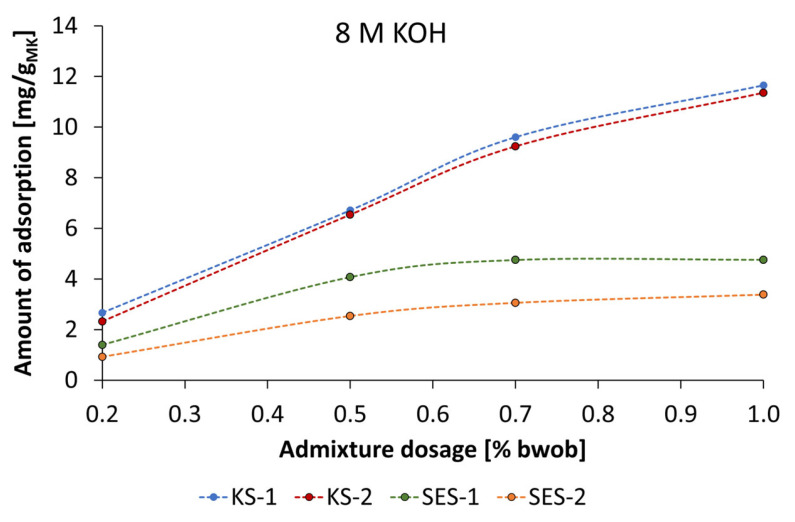
Adsorption isotherms of anionic and cationic starch admixtures of geopolymer pastes activated by 8 mol/L KOH.

Pastes activated by 8 mol/L KOH (Figure 20), the adsorption isotherms of SES-1 and SES-2 followed the Langmuir type but at different values of adsorption. Both admixture samples reached the adsorption plateau at a dosage of 0.7% bwob; however, the amount of adsorption for SES-1 is higher by a factor of 1.5. The cationic starch samples produce a similar adsorption isotherm, where a linear increase occurs until 0.7% bwob, and then the isotherm increases by a smaller adsorption rate. An adsorption plateau was not observed at the investigated admixture concentrations. These results fit well with dispersing performance, where the spread flow increased at dosages up to 1.5% bwob.

**Figure 21:**
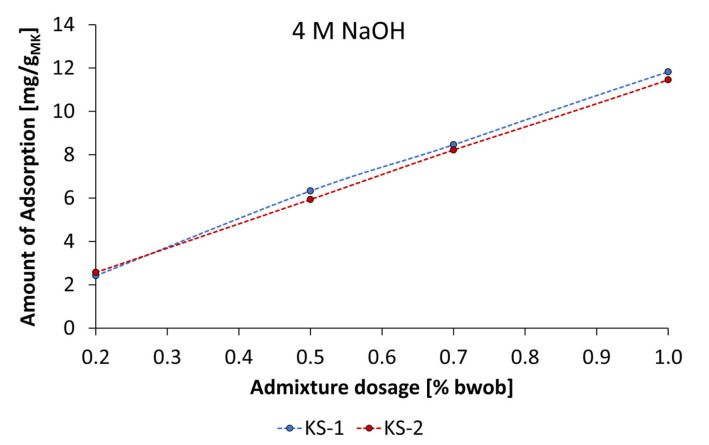
Adsorption isotherms of cationic starch admixtures on geopolymer pastes activated by 4 mol/L NaOH.

**Figure 22:**
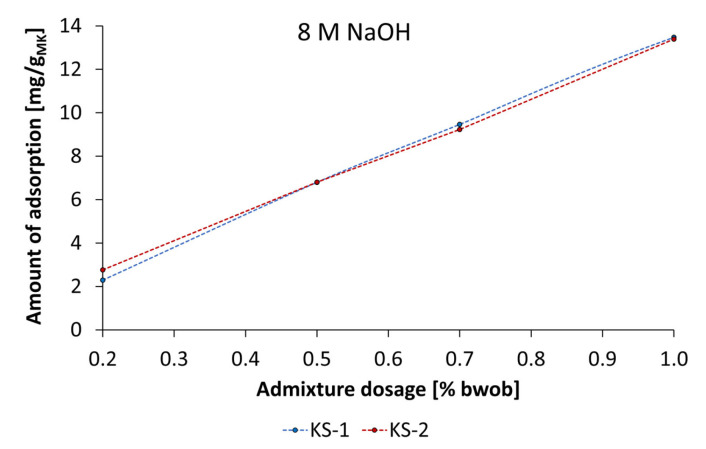
Adsorption isotherms of cationic starch admixtures on geopolymer pastes activated by 8 mol/L NaOH.

The adsorption isotherms of cationic starch admixtures in geopolymer pastes activated by 4 mol/L and 8 mol/L are shown in Figure 21 and Figure 22. In general, KS-1 and KS-2 produce Freundlich-type isotherms, which are described by a linear increase in adsorption by increasing admixture dosage. This corresponds to a mini slump, whereby increasing the concentration of the admixture increased the dispersing performance. The higher dispersing performance in 8 mol/L NaOH can be explained by a slightly higher amount of adsorption by a factor of 1.15. Generally, the difference in the amount of adsorption of starch admixtures by using KOH and NaOH in pastes could be explained by the significantly higher solubility of metakaolin activated by NaOH. As a result of the dissolution of the particles, larger surfaces may be available for the adsorption of admixtures.

### 3.5. Influence of Starch Admixtures on Geopolymer Reaction

**Figure 23:**
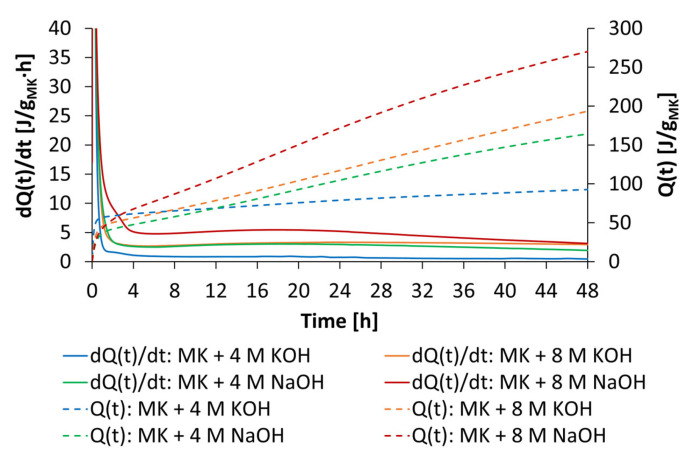
Heat rate and cumulative heat release of geopolymer pastes activated by different activator types (KOH and NaOH) and concentrations (4 mol/L and 8 mol/L).

Due to the adsorptive effect of starch admixtures, the influence on reaction kinetics as well as the reaction degree was investigated by calorimetric studies. Therefore, at first the heat rate and cumulative heat release in dependence of the type and concentration of the alkaline activator were measured. Figure 23 shows that the heat rate increased by increasing activator concentration from 4 mol/L to 8 mol/L for both types of activators. The lowest heat rate and cumulative heat release of pastes were detected using 4 mol/L of KOH, and the highest ones were reached using 8 mol/L of NaOH. Interestingly, the use of 8 mol/L KOH and 4 mol/L NaOH supplied comparable values in heat rate.

The comparison of heat rate and cumulative heat release as well as the influence of the synthesized starch admixtures at specific times of 8 h, 24 h, and 48 h is given in Table 6. In pastes activated by 4 mol/L of KOH, the anionic as well as the cationic starch admixture shows no significant influence on reaction kinetics and degree of reaction in the period under consideration. Furthermore, by using 8 mol/L of KOH, the reaction degree differs slightly, which corresponds to negligible differences in cumulative heat release after 48 h of 10 J/g_MK_. The same tendency in reaction degree and heat rate was found for pastes activated by 4 mol/L and 8 mol/L of NaOH. The differences in heat rate and cumulative heat release at the investigated times are negligibly low, which indicates no significant influence on reaction kinetics by the cationic starch admixtures.

### 3.6. Influence of Starch Admixtures on Mechanical Properties and Bulk Densities of Geopolymer Fine Mortars

The bulk density values of the geopolymer mortars with and without anionic and cationic starch admixtures are given in Table 7. In pastes activated by 4 mol/L of KOH, the bulk density increased from 1.38 g/cm^3^ to approx. 1.5 g/cm^3^ due to the reduction of the l/s ratio. The same effect can be seen by the use of 8 mol/L of KOH. The bulk density increased from 1.46 g/cm^3^ up to approx. 1.64 g/cm^3^. Interestingly, the bulk density of pastes activated by NaOH is just minimally affected by reduction of the l/s value and the use of the starch admixtures.

**Figure 24:**
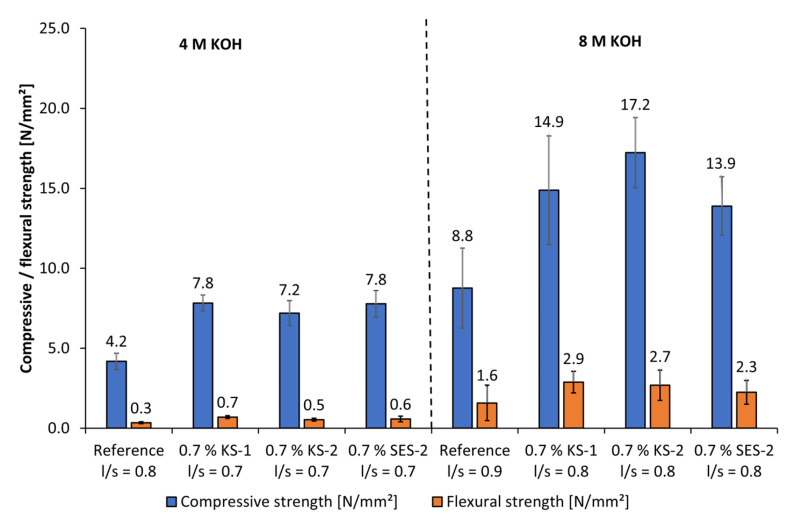
Mechanical properties of geopolymer fine mortars with anionic and cationic starch admixtures activated by 4 mol/L and 8 mol/L KOH.

The flexural strength as well as the compressive strength of geopolymer fine mortars activated by 4 mol/L and 8 mol/L of KOH are presented in Figure 24. By the use of anionic and cationic starch admixtures (0.7% bwob), the l/s ratio could be reduced by the factor of 0.1 for both activator concentrations at similar workability. This led to increased flexural strengths and compressive strengths at both concentrations of KOH. Furthermore, the mechanical properties increased by increasing the concentration from 4 mol/L to 8 mol/L of KOH. These results fit well with the solubility investigations, where higher dissolved silicon and aluminum values were reached by increasing the concentration of KOH activators. Accordingly, it was expected that the geopolymer mortars mixed by 4 mol/L KOH did not reach high strength values. The highest compressive strength of 17.2 MPa was reached by the fine mortar admixed with KS-2 and 8 mol/L of KOH, which also caused the highest dispersing performance.

**Figure 25:**
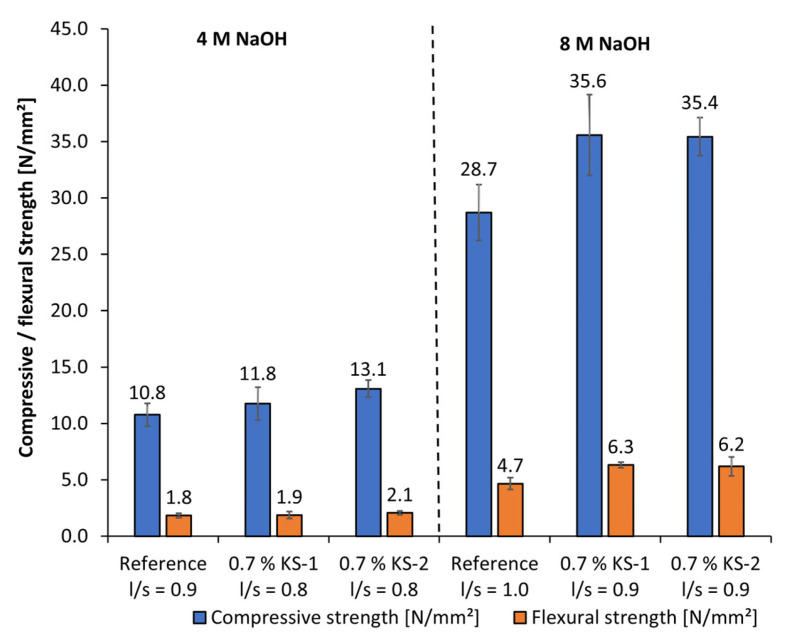
Mechanical properties of geopolymer fine mortars with cationic starch admixtures activated by 4 mol/L and 8 mol/L NaOH.

The geopolymer fine mortars activated by 8 mol/L of NaOH reached quite higher compressive strengths in the range of approx. 29 MPa to 36 MPa than those activated by 4 mol/L of NaOH (Figure 25). Due to the significantly higher solubility of silicon and aluminum using NaOH, the flexural strengths and compressive strengths take significantly higher values than using KOH as an activator at all concentrations. Just the cationic starch admixtures were admixed in geopolymer fine mortars using NaOH activator because the anionic starch admixtures show no liquefying effect. Both cationic starch admixtures cause an increase in flexural strength and compressive strength because of the reduction of the l/s ratio at similar workability. In general, using cationic starch admixtures, a reduction of the l/s ratio is possible, which leads to an increase in compressive and flexural strength. Furthermore, high alkaline activators are necessary to reach viable mechanical properties.

## 4. Conclusions

This study provides new insights into the interaction of PCE and modified starch admixtures in different alkaline solutions used for geopolymer preparation. The investigations aimed to determine the conformation of PCE and starch admixtures in KOH and NaOH solutions depending on activator concentration. The dispersing performance and adsorption behavior in pastes prepared by metakaolin and alkaline activators, as well as the influence on reaction kinetics and degree depending on the type and concentration of alkaline activator, were recorded.

The following conclusions can be drawn based on the results obtained:

1. At all types of activators and their concentrations, the metakaolin dissolved congruently, and the amount of dissolved aluminum and silicon depends strongly on the concentration of the activator. Furthermore, in the NaOH activator, the dissolution process of silicon and aluminum species occurs faster and in higher amounts.
2. The PCE superplasticizers show coiling and formation of insoluble aggregates from concentrations of 3 mol/L in both KOH and NaOH. Therefore, PCE superplasticizers are globally incompatible superplasticizers for geopolymer binders activated by high alkaline solutions higher than 3 mol/L.
3. Anionic and cationic starch admixtures were synthesized with high and low charge density, which are soluble in each type and concentration of activator and show no coiling effects or formation of insoluble aggregates.
4. The anionic starch admixture shows lower dispersing performance in KOH than cationic ones and no dispersing effect in NaOH independent of concentration.
5. The cationic starch admixtures are more effective in both types of activators, where especially the high-charged sample reached the highest dispersing performance.
6. The mechanism of action is obviously based on adsorption of dissolved metakaolin particles. The adsorption behavior can be described by Langmuir-type isotherms for the anionic starch admixtures in pastes prepared by KOH and the Freundlich-type isotherm for cationic starch admixture in pastes activated by NaOH at the investigated concentrations. Furthermore, the amount of adsorbed cationic starch admixture is remarkably higher at all concentrations.
7. The reaction degree and reaction kinetics of geopolymer pastes depend strongly on activator concentration, where 8 mol/L caused the highest heat rate and cumulative heat release. This fact is consistent with solubility investigations, where at the highest activator concentration, the highest amounts of silicon and aluminum were released and can react to an alumino-silicate network. Both types of starch admixture caused no significant effect on reaction kinetics and reaction degree at both activator types and concentrations of 4 mol/L and 8 mol/L.
8. Only at high concentrations of the examined alkaline activators were viable mechanical properties reached. Therefore, classical PCE superplasticizers are not suitable admixtures for such materials. In contrast, especially cationic starch admixtures are able to disperse the highly alkali-activated metakaolin. Therefore, the l/s ratio could be reduced, which leads to an increase in flexural and compressive strengths.

In general, the fluidizing effect of the synthesized starch admixture is still lower in geopolymer pastes than in cementitious systems [29,30,48] and not comparable to the dispersing performance of superplasticizers in OPC pastes. In contrast, due to the fact that currently no effective admixtures are available for alkali-activated metakaolin-based geopolymers, the modified starch admixtures could be an interesting starting point for further developments. Future investigations are necessary to discover the influence of cationic starch admixtures on durability and chemical resistance against aggressive media. Furthermore, the synthesis of cationic starch admixtures can be improved by the implementation of side chains to achieve steric repulsion, which can increase dispersing performance. The particle–polymer interaction of the synthesized starch admixtures and 2:1 clay-like illite- or smectite-dominated clays will be an interesting future research topic due to their worldwide occurrence.

## Figures and Tables

**Table 1:** Mineralogical and chemical composition of the used metakaolin and its physical parameters.

Oxides [wt.-%]	SiO_2_	Al_2_O_3_	Fe_2_O_3_	CaO	MgO	TiO_2_	K_2_O	Na_2_O	SO_3_	P_2_O_5_	DL ^1^	LOI ^2^
	52.0	41.4	0.6	0.0	0.1	0.93	0.3	0.03	0.1	0.1	0.4	3.7
Phases [%]	Kaolinite		Anatase		Quartz		Calcite		Amorphous			
	23.9 ± 1.5		0.9 ± 0.2		2.7 ± 0.3		0.9 ± 0.4		71.6 ± 1.5			
Particle size [µm]	d_10_ 0.8		d_50_ 8.19		d_90_ 20.2							
BET [m^2^/g]	11.5											
Puzzolanic reactivity	R^3^-Test (7 d, 40 °C)					Chapelle Test						
	Q = 959.3 J/g_MK_					C = 1250 mgCaO/g_MK_						

^1^ Drying loss at 105 °C. ^2^ Loss of ignition at 950 °C.

**Table 2:** Chemical parameters of the used NaOH and KOH activators by [33,34].

KOH Solutions			
Molarity [mol/L]	1 mol/L	4 mol/L	8 mol/L
Molality [mol/kg]	0.99	3.5	6
pH value [-]	13.89	14.61	15.28
Activity coefficient [-]	0.733	1.184	2.18
Activity [mol/L]	0.733	4.736	17.44
**NaOH Solutions**			
Molarity [mol/L]	1 mol/L	4 mol/L	8 mol/L
Molality [mol/kg]	0.99	3.5	6
pH value [-]	14.01	14.53	15.04
Activity coefficient [-]	0.668	0.847	1.302
Activity [mol/L]	0.668	3.388	10.416

**Table 3:** Molecular weight, polydispersity, and anionic charge density of PCE-based superplasticizer.

Parameter	HPEG	IPEG	MPEG
Mn [g/mol]	8286	7388	7984
Mw [g/mol]	13,390	11,460	17,340
PDI [-]	1.62	1.55	2.17
Anionic charge density in H_2_O ^1^ [µeq/g]	6989 ± 18 ^2^	9162 ± 10 ^2^	9871 ± 33 ^2^

^1^ Deionized water. ^2^ Titrant: 0.001 N polydiallyldimethylammoniumchloride (Poly-DADMAC).

**Table 4:** Molecular weight, polydispersity, and anionic and cationic charge density of the anionic and cationic starch admixtures.

Parameter	Basic Starch	SES-1	SES-2	KS-1	KS-2
Mn [g/mol]	9629	17,880	36,860	19,670	34,890
Mw [g/mol]	38,890	41,410	39,620	31,270	37,500
PDI [-]	4.04	2.32	1.08	1.59	1.08
Anionic charge density in H_2_O ^1^ [µeq/g]	-	946 ± 1 ^2^	2422 ± 1 ^2^	-	-
Cationic charge density in H_2_O ^1^ [µeq/g]	-	-	-	1017 ± 1 ^3^	2142 ± 2 ^3^

^1^ Deionized water. ^2^ Titrant: 0.001 N polydiallyldimethylammoniumchloride (Poly-DADMAC). ^3^ Titrant: 0.001 N sodium polyethylene sulfonic acid (PES-Na).

**Table 5:** Mineralogical and chemical composition of the used granite crushed sand and its physical parameters (particle size and BET surface).

Oxides [wt.-%]	SiO_2_	Al_2_O_3_	Fe_2_O_3_	CaO	MgO	TiO_2_	MnO	K_2_O	Na_2_O	SO_3_	P_2_O_5_	DL ^1^	LOI ^2^
	67.3	14.8	3.4	2.4	1.4	0.29	0.18	4.27	3.29	0.4	0.51	0.1	1.6
Phases [%]	Chlorite		Muscovite		Orthoclase	Albite		Andesine		Pyrite		Quartz	
	3.5 ± 0.9		11.8 ± 1.5		21.6 ± 1.5	28.4 ± 2.4		6.7 ± 2.3		0.3 ± 0.1		27.7 ± 1.0	
Particle size [µm]	d_10_		d_50_		d_90_								
	63		250		500								
BET [m^2^/g]	2.5												

^1^ Drying loss at 105 °C. ^2^ Loss of ignition at 950 °C.

**Table 6:** Heat rate and cumulative heat at 8 h (t_1_), 24 h (t_2_), and 48 h (t_3_) of the geopolymer samples admixed with 0.7% bwob of the starch admixtures and KOH.

Sample	dQ(t_1_)/dt [J/g_MK_∙h]	dQ(t_2_)/dt [J/g_MK_∙h]	dQ(t_3_)/dt [J/g_MK_∙h]	Q(t_1_) [J/g_MK_]	Q(t_2_) [J/g_MK_]	Q(t_3_) [J/g_MK_]
MK + 4 M KOH	1.2	1.2	0.9	46.8	66.4	92.1
0.7% SES-1	1.3	1.3	1.0	42.3	63.9	92.0
0.7% SES-2	1.3	1.3	1.0	41.1	61.6	88.6
0.7% KS-1	1.3	1.3	1.0	49.4	65.4	92.8
0.7% KS-2	1.3	1.3	1.0	47.2	62.5	89.1
MK + 8 M KOH	3.0	3.3	2.9	78.7	117.3	193.4
0.7% SES-1	3.0	3.3	2.9	84.9	123.0	197.9
0.7% SES-2	3.1	3.3	2.9	89.4	128.5	204.4
0.7% KS-1	3.0	3.3	2.9	84.3	122.2	196.7
0.7% KS-2	3.0	3.3	2.9	86.4	124.3	199.4
MK + 4 M NaOH	2.9	3.0	1.9	68.9	104.8	164.2
0.7% KS-1	2.8	2.9	1,9	66.4	101.8	159.7
0.7% KS-2	2.9	3.0	1.9	68.0	103.8	162.5
MK + 8 M NaOH	5.2	5.2	3.1	107.2	171.7	270.1
0.7% KS-1	5.3	5.3	3.1	109.0	174.1	272.5
0.7% KS-2	5.1	5.1	3.1	107.0	170.4	267.9

**Table 7:** Bulk densities of the geopolymer mortars admixed with 0.7% bwob of the starch admixtures.

Sample	Bulk Density [g/cm^3^]	Sample	Bulk Density [g/cm^3^]
4 M KOH		8 M KOH	
Reference, l/s = 0.8	1.38 ± 0.06	Reference, l/s = 0.9	1.46 ± 0.08
0.7% KS-1, l/s = 0.7	1.53 ± 0.05	0.7% KS-1, l/s = 0.8	1.64 ± 0.10
0.7% KS-2, l/s = 0.7	1.50 ± 0.07	0.7% KS-2, l/s = 0.8	1.57 ± 0.04
0.7% SES-2, l/s = 0.7	1.50 ± 0.07	0.7% SES-2, l/s = 0.8	1.55 ± 0.12
4 M NaOH		8 M NaOH	
Reference, l/s = 0.9	1.42 ± 0.04	Reference, l/s = 1.0	1.48 ± 0.05
0.7% KS-1, l/s = 0.8	1.44 ± 0.06	0.7% KS-1, l/s = 0.9	1.57 ± 0.03
0.7% KS-2, l/s = 0.8	1.48 ± 0.02	0.7% KS-2, l/s = 0.9	1.54 ± 0.07

## Data Availability

The original contributions presented in this study are included in the article. Further inquiries can be directed to the corresponding author.

## References

[1] Benhelal E., Zahedi G., Shamsaei E., Bahadori A. (2013). Global strategies and potentials to curb CO2 emissions in cement industry. J. Clean. Prod..

[2] Pol Segura I., Ranjbar N., Juul Damø A., Skaarup Jensen L., Canut M., Arendt Jensen P. (2023). A review: Alkali-activated cement and concrete production technologies available in the industry. Heliyon.

[3] Davidovits J. (2020). Geopolymer Chemistry and Applications.

[4] Kaps C., Hohmann M., Partschefeld S. (2012). Zur Reaktivität von Säure-/Base-aktivierten Metatonen in Spezialbindemitteln. Proceedings of the ibausil—International Conference on Building Materials.

[5] Agashua L.O., Arum C., Oluyemi-Ayibiowu B.D., Ikumapayi C.M. (2024). A systematic review of geopolymer materials: Innovations, prevailing constraints and resolutions. Sinergi.

[6] Nasir M., Mahmood A.H., Bahraq A.A. (2024). History, recent progress, and future challenges of alkali-activated binders—An overview. Constr. Build. Mater..

[7] Provis J.L., Bernal S.A. (2014). Geopolymers and Related Alkali-Activated Materials. Annu. Rev. Mater. Res..

[8] Liew Y.-M., Heah C.-Y., Mohd Mustafa A.B., Kamarudin H. (2016). Structure and properties of clay-based geopolymer cements: A review. Prog. Mater. Sci..

[9] Nodehi M., Taghvaee V.M. (2022). Alkali-Activated Materials and Geopolymer: A Review of Common Precursors and Activators Addressing Circular Economy. Circ. Econ. Sust..

[10] Farooq F., Jin X., Faisal Javed M., Akbar A., Izhar Shah M., Aslam F., Alyousef R. (2021). Geopolymer concrete as sustainable material: A state of the art review. Constr. Build. Mater..

[11] Anudeep P., Reddy M.A.K., Khed V.C., Adamu M., Varalakshmi M., Ibrahim Y.E., Ahmed O.S. (2024). Effect of superplasticizer in geopolymer and alkali-activated cement mortar/concrete: A review. Rev. Adv. Mater. Sci..

[12] Plank J., Sakai E., Miao C.W., Yu C., Hong J.X. (2015). Chemical admixtures—Chemistry, applications and their impact on concrete microstructure and durability. Cem. Concr. Res..

[13] Lei L., Palacios M., Plank J., Jeknavorian A.A. (2022). Interaction between polycarboxylate superplasticizers and non-calcined clays and calcined clays: A review. Cem. Concr. Res..

[14] Ng S., Plank J. (2012). Interaction mechanisms between Na montmorillonite clay and MPEG-based polycarboxylate superplasticizers. Cem. Concr. Res..

[15] Marchon D., Sulser U., Eberhardt A., Flatt R.J. (2013). Molecular design of comb-shaped polycarboxylate dispersants for environmentally friendly concrete. Soft Matter.

[16] Chen X., Jin B., Suraneni P. (2024). Understanding the dissolution of metakaolin in sodium hydroxide solutions. Mater. Struct..

[17] Weng L., Sagoe-Crentsil K. (2007). Dissolution processes, hydrolysis and condensation reactions during geopolymer synthesis: Part I—Low Si/Al ratio systems. J. Mater. Sci..

[18] Moghul S., Zunino F., Flatt R.J. (2025). Flow loss in superplasticized limestone calcined clay cement. J. Am. Ceram. Soc..

[19] Sposito R., Beuntner N., Thienel K.-C. (2020). Characteristics of components in calcined clays and their influence on the efficiency of superplasticizers. Cem. Concr. Compos..

[20] Schmid M., Plank J. (2020). Dispersing performance of different kinds of polycarboxylate (PCE) superplasticizers in cement blended with a calcined clay. Constr. Build. Mater..

[21] Chen J., Plank J. (2025). Which factors impact the effectiveness of PCEs in alkali-activated slag cements?. Cem. Concr. Res..

[22] Tutal A., Partschefeld S., Schneider J., Osburg A. (2020). Effects of Bio-Based Plasticizers, Made from Starch, on the Properties of Fresh and Hardened Metakaolin-Geopolymer Mortar: Basic Investigations. Clays Clay Miner..

[23] Palacios M., Houst Y.F., Bowen P., Puertas F. (2009). Adsorption of superplasticizer admixtures on alkali-activated slag pastes. Cem. Concr. Res..

[24] Wang Y.-S., Alrefaei Y., Dai J.-G. (2020). Influence of coal fly ash on the early performance enhancement and formation mechanisms of silico-aluminophosphate geopolymer. Cem. Concr. Res..

[25] Kim Y.J., Choi S., Oh S.R. (2024). The Effects of Ester and Ether Polycarboxylate Superplasticizers on the Fluidity and Setting Behavior of Alkali-Activated Slag Paste. Materials.

[26] Palacios M., Puertas F. (2004). Stability of superplasticizer and shrinkage-reducing admixtures Stability of superplasticizer and shrinkage-reducing admixtures in high basic media. Mater. Constr..

[27] Palacios M., Puertas F. (2005). Effect of superplasticizer and shrinkage-reducing admixtures on alkali-activated slag pastes and mortars. Cem. Concr. Res..

[28] Favier A., Hot J., Habert G., Roussel N., d’Espinose de Lacaillerie J.-B. (2014). Flow properties of MK-based geopolymer pastes. A comparative study with standard Portland cement pastes. Soft Matter.

[29] Vieira M.C., Klemm D., Einfeldt L., Albrecht G. (2005). Dispersing agents for cement based on modified polysaccharides. Cem. Concr. Res..

[30] Crépy L., Petit J.-Y., Wirquin E., Martin P., Joly N. (2014). Synthesis and evaluation of starch-based polymers as potential dispersants in cement pastes and self leveling compounds. Cem. Concr. Compos..

[31] (2012). Betonzusatzstoffe—Metakaolin—Festlegung und Konformitätskriterien: Addition pour béton Hydraulique—Métakaolin—Spécifications et Critères de Conformité.

[32] Suraneni P. (2021). Recent developments in reactivity testing of supplementary cementitious materials. RILEM Tech. Lett..

[33] Hamer W.J., Wu Y.-C. (1972). Osmotic Coefficients and Mean Activity Coefficients of Uni-univalent Electrolytes in Water at 25 °C. J. Phys. Chem. Ref. Data.

[34] Hausmann J.N., Traynor B., Myers R.J., Driess M., Menezes P.W. (2021). The pH of Aqueous NaOH/KOH Solutions: A Critical and Non-trivial Parameter for Electrocatalysis. ACS Energy Lett..

[35] Matthys S., Proia A. (2023). Proceedings of the DuRSAAM 2023 Symposium on Advancing Alkali-Activated Materials.

[36] Tan Z., Bernal S.A., Provis J.L. (2017). Reproducible mini-slump test procedure for measuring the yield stress of cementitious pastes. Mater. Struct..

[37] Perche F. (2004). Adsorption de Polycarboxylates et de Lignosulfonates sur Poudre Modèle et Ciments. Ph.D. Thesis.

[38] Provis J.L., van Deventer J.S.J. (2014). Alkali Activated Materials.

[39] Zhang Y., Chan H.-K., Han Z., Lei L. (2024). Why do conventional MAA-MPEG PCEs not work in alkali-activated slag systems?. Cem. Concr. Res..

[40] Werling N., Kaltenbach J., Weidler P.G., Schuhmann R., Dehn F., Emmerich K. (2022). Solubility of Calcined Kaolinite, Montmorillonite, and Illite in High Molar NaOH and Suitability as Precursors for Geopolymers. Clays Clay Miner..

[41] Werling N., Dehn F., Krause F., Steudel A., Schuhmann R., Emmerich K. (2020). Solubility of precursors and carbonation of waterglass-free geopolymers. Clays Clay Miner..

[42] Dathe F., Overmann S., Koenig A., Dehn F. (2024). The Role of Water Content and Binder to Aggregate Ratio on the Performance of Metakaolin-Based Geopolymer Mortars. Minerals.

[43] Lange A., Plank J. (2016). Contribution of non-adsorbing polymers to cement dispersion. Cem. Concr. Res..

[44] Wu B., Gu L., Chun B.-W., Kuhl T.L. (2022). Adsorption and interaction forces of commercial Poly (naphthalene sulfonate) (PNS) and Poly (carboxylate ether) (PCE) polyelectrolytes with negatively charged surfaces in monovalent and divalent electrolytes. Colloids Surf. A Physicochem. Eng. Asp..

[45] Alrefaei Y., Dai J.-G. (2022). Effects of delayed addition of polycarboxylate ether on one-part alkali-activated fly ash/slag pastes: Adsorption, reaction kinetics, and rheology. Constr. Build. Mater..

[46] Freundlich H. (1907). Über die Adsorption in Lösungen. Z. Für Phys. Chem..

[47] Physical Chemistry [Medienkombination].

[48] Partschefeld S., Osburg A. (2020). Synthesis of dispersing agents from starch—Influence on rheological properties and early age hydration of OPC. Constr. Build. Mater..

